# The Mediating Role of Executive Function in the Association Between Warm Parenting and Children’s Problem Behaviors

**DOI:** 10.3390/children13020224

**Published:** 2026-02-05

**Authors:** Hyungmin Lee

**Affiliations:** Department of Wellness Industry Convergence, Center for Smile Aging Research, Hankyong National University, Anseong-si 17579, Republic of Korea; miso2child@hknu.ac.kr; Tel.: +82-31-670-5235; Fax: +82-31-670-5239

**Keywords:** warm parenting, executive function, problem behaviors, early school-age children, mediation

## Abstract

**Highlights:**

**What are the main findings?**

- Results reveal that maternal warm parenting is directly associated with fewer problem behaviors in children.
- Findings also reveal the mediating role of executive function (EF); maternal warmth was strongly associated with children’s EF skills, and EF, in turn, predicted children’s behavioral outcomes.

**What are the implications of the main findings?**

- The findings have meaningful implications for prevention and intervention efforts addressing aspects related to parenting and children’s EF and problem behaviors.
- The results can inform early intervention efforts aimed at strengthening regulatory capacities and promoting adaptive functioning during the foundational years of schooling.

**Abstract:**

**Background/Objectives**: The present study explored the extent to which children’s executive function (EF) mediates the relationship between maternal warm parenting and children’s problem behaviors during the early school-age years. **Methods**: Using data from a nationally representative sample of 1516 mother–child dyads participating in the Panel Study on Korean Children, the analysis drew on parent-reported measures collected through standardized questionnaires. **Results**: Structural equation modeling indicated that warm, responsive maternal parenting contributed to lower levels of children’s problem behaviors both directly and indirectly by fostering stronger EF skills. **Conclusions**: The findings highlight the developmental importance of warm parenting practices and identify children’s EF as a central mechanism through which early caregiving environments promote behavioral adjustment.

## 1. Introduction

The transition into formal schooling is a critical developmental juncture during which children are required to meet increasingly complex academic expectations, adapt to structured classroom routines, and navigate broader peer networks. Unlike early childhood settings, elementary school environments place greater demands on children’s ability to sustain attention, inhibit impulsive responses, comply with explicit rules, and manage multifaceted learning tasks that require persistence and goal-directed behavior. These heightened expectations underscore the centrality of executive function (EF)—a constellation of higher-order cognitive processes, including inhibitory control, working memory, and cognitive flexibility—which enables children to regulate behavior in situations involving novelty, challenge, or cognitive load [1]. As children encounter these academic and socioemotional demands, EF becomes a foundational mechanism supporting school readiness, learning engagement, and overall adjustment.

Extensive empirical research indicates that deficits in EF are associated with elevated levels of both externalizing behaviors (e.g., aggression, impulsivity, hyperactivity) and internalizing difficulties (e.g., anxiety, social withdrawal, depression) throughout childhood. Further, meta-analytic evidence demonstrates that children with externalizing behavior problems exhibit deficits in executive functioning, particularly in inhibitory control [2]. Prospective evidence indicates that lower EF skills in early childhood are associated with increases in internalizing problems, including anxiety and withdrawal, over time [3]. From a developmental perspective, early caregiving environments characterized by warmth and sensitive scaffolding play a critical role in shaping EF by supporting children’s gradual transition from external regulation to self-regulation [4]. In this regard, EF provides children with essential cognitive resources for managing emotional reactivity, navigating peer interactions, and sustaining adaptive engagement in classroom contexts.

Warm parenting provides an emotionally secure foundation that supports regulatory development and is associated with secure attachment, emotion regulation, and fewer behavioral problems [5,6,7,8,9], whereas harsh or inconsistent parenting practices are linked to elevated risks of both externalizing and internalizing symptoms [10,11,12]. Combined, these findings underscore warm caregiving as a salient socioemotional protective factor and highlight the relative paucity of empirical research from the Korean context.

Early emerging behavioral challenges can increase risks of maladaptive developmental trajectories and later psychopathology [13]. In particular, externalizing behaviors display notable developmental stability and have been shown to undermine children’s peer relationships, classroom engagement, and socioemotional competence [14]. Various familial stressors—such as parenting stress, parental depression, and marital conflict—have been identified as predictors of behavioral maladjustment [15,16,17,18,19,20]. Nevertheless, warm and supportive parenting consistently emerges as a robust buffer against behavioral risk across diverse samples and developmental periods [21,22,23,24,25,26].

EF—encompassing inhibitory control, working memory, and cognitive flexibility—supports behavioral regulation in response to situational demands [27]. Deficits in EF have been associated with elevated externalizing behaviors and, to a lesser extent, internalizing symptoms [28,29,30,31]. Longitudinal studies indicate that early inhibitory control predicts future behavioral outcomes [32,33,34,35].

EF development reflects an ongoing interaction between neurobiological maturation and environmental influences [36,37,38]. Within this context, parenting processes characterized by scaffolding, cognitive stimulation, sensitivity, and balanced behavioral control have been consistently identified as key contributors to the growth of EF [39]. Scaffolding refers to parents’ intentional efforts to provide verbal or nonverbal support when children encounter challenging tasks, thereby enabling the latter to articulate their ideas and engage in problem-solving activities. Emotionally supportive parenting provides children with everyday opportunities to develop and exercise regulatory and attentional skills [40]. In addition, parental sensitivity and responsiveness contribute to the internalization of self-regulatory strategies, while appropriate parenting practices facilitate the development of desirable self-control in children [41].

Evidence from Korean studies aligns with this literature, indicating that maternal warmth is associated with children’s delayed gratification, planning abilities, inhibitory control, and attentional flexibility [42,43]. Recent longitudinal analyses also indicate that warm, supportive caregiving reduces EF-related difficulties [44,45].

Despite extensive theoretical and empirical evidence linking parenting, EF, and behavioral outcomes, relatively few studies have examined EF as a mediating mechanism connecting warm parenting to behavioral adjustment among early school-aged children, particularly in Korea. Existing work has focused on younger preschool populations [46], children living in rural poverty [47], older adolescents [5], and/or clinically referred groups such as children with ADHD [46,48].

In these international studies, longitudinal evidence consistently indicates that positive parenting practices are associated with the development of children’s self-regulatory capacities, which in turn contribute to subsequent reductions in externalizing problem behaviors. In studies involving clinical populations, the relative influence of symptom severity on behavioral outcomes has been revealed to vary across conditions; however, these findings also highlight the importance of lower levels of parenting stress and the presence of supportive and adaptive parenting practices within the family context. As expectations for behavioral regulation intensify during the early school years, understanding EF as a cognitive mechanism that links caregiving environments to children’s behavioral adjustment is both theoretically compelling and practically consequential.

With EF assessed for the first time in the nationally representative Panel Study on Korean Children (PSKC), the present study leverages a unique opportunity to examine developmental pathways underlying children’s socioemotional adjustment within a large-scale, population-based context. By drawing on a nationally representative sample, this study extends prior research that has predominantly relied on smaller or clinically focused samples and provides broader insight into the role of EF as a developmental mechanism in normative populations.

**Figure 1:**
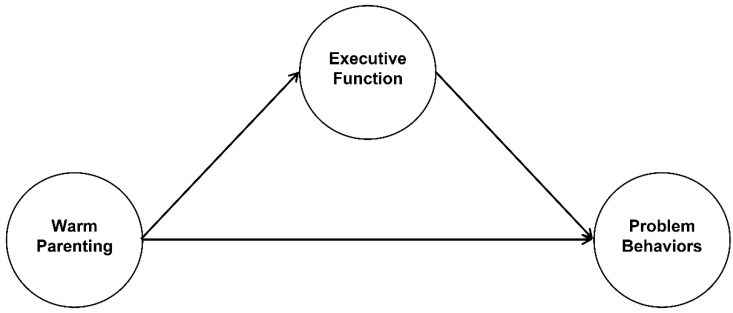
Structural model illustrating the mediating role of executive function.

In particular, this study investigates whether EF mediates the association between maternal warm parenting and children’s problem behaviors, thereby contributing to a more comprehensive understanding of the cognitive–affective processes through which caregiving shapes behavioral development. Findings from this research may inform early intervention efforts aimed at strengthening regulatory capacities and promoting adaptive functioning during the foundational years of schooling. Accordingly, a research model was developed to examine the proposed relationships among the study variables (Figure 1).

## 2. Materials and Methods

### 2.1. Participants

Data for the present study were drawn from Wave 8 of the PSKC, a long-term study to track the growth process of Korean children, conducted by the Korea Institute of Child Care and Education (KICCE). Detailed information about the PSKC and procedures for data access are publicly available on the KICCE website [49]. Access to its nationally representative longitudinal dataset was granted following KICCE’s institutional approval procedures, and all analyses were conducted in accordance with the data use guidelines. After removing cases with incomplete or invalid responses, a final sample of 1516 mother–child dyads was retained for analysis.

Table 1 summarizes the descriptive characteristics of the participants. Mothers’ educational attainment was generally high, with the majority having completed at least a high school education and a substantial proportion holding a four-year university degree. Mothers’ employment status also varied across occupational categories, including managerial or professional and clerical positions, while a considerable proportion were unemployed or engaged in other forms of economic activity.

As for children’s characteristics, 51.1% of the children were boys (n = 775) and 48.9% were girls (n = 741). Children ranged in age from 84 to 92 months, with the largest proportion (46.0%, n = 697) clustered within the 87–88-month range. Regarding birth order, first-born children comprised the largest group (46.6%, n = 707), followed by second-born children (42.1%, n = 638) and those who were third-born or later (11.3%, n = 171).

### 2.2. Measures

#### 2.2.1. Maternal Warm Parenting

Maternal warm parenting was assessed using the scale originally developed by Cho et al. [50]. This instrument consists of eight items capturing affectionate, responsive, and supportive parenting behaviors. Mothers rated each item on a 5-point Likert scale ranging from 1 (=“not at all true”) to 5 (=“very true”). Representative items include respecting the child’s opinions and showing interest in the child’s activities. Composite scores were calculated, where higher values reflected greater levels of warm parenting. Internal consistency for the present sample was high (Cronbach’s α = 0.87).

#### 2.2.2. Children’s EF

Children’s EF was measured using the instrument validated by Song [43]. The scale consists of 40 items assessing difficulties in core EF-related domains, including difficulties in planning–organization, behavioral control, emotional control, and inattention.

Mothers rated each item on a 3-point Likert scale from 1 (=“not at all true”) to 3 (=“often true”). The items capture everyday manifestations of EF-related difficulties, such as delaying task initiation, difficulty remaining seated, heightened emotional reactivity to minor situations, and frequent loss of personal belongings.

Higher scores indicate greater EF difficulties. Reliability coefficients (Cronbach’s α) for the four subscales were 0.88 (planning–organization difficulties), 0.86 (behavioral control difficulties), 0.90 (emotional control difficulties), and 0.89 (inattention), demonstrating strong internal consistency. The total EF score also demonstrated excellent reliability (Cronbach’s α = 0.94).

#### 2.2.3. Children’s Problem Behaviors

Children’s problem behaviors were assessed using the Korean version of the Child Behavior Checklist for Ages 6–18 (CBCL 6–18) [51], focusing on the Internalizing Problems and Externalizing Problems subscales.

Internalizing problems capture overly controlled behaviors such as withdrawal, anxiety, and somatic complaints, whereas externalizing problems reflect undercontrolled behaviors including aggression, rule-breaking, and overt conduct issues.

The internalizing and externalizing subscales comprise 32 and 35 items, respectively. Mothers rated each item using a 3-point Likert scale (0 = “not true,” 1 = “somewhat or sometimes true,” and 2 = “very true or often true”). Internal consistency was acceptable, with Cronbach’s α = 0.75 and 0.81 for internalizing and externalizing problems, respectively.

### 2.3. Data Analysis

Descriptive statistics were computed and frequency analyses were conducted to examine the sociodemographic characteristics of the sample. For all major study variables, minimum and maximum values, means, standard deviations, skewness, and kurtosis were calculated.

Bivariate associations between maternal warm parenting, children’s EF, and children’s problem behaviors were examined using Pearson’s correlation coefficients. Structural equation modeling (SEM) was conducted using AMOS 24.0 (IBM Corp., Armonk, NY, USA), and model fit was evaluated using standard model fit indices (chi-square statistic [χ^2^], normed fit index [NFI], comparative fit index [CFI], and root mean square error of approximation [RMSEA]). Indirect (mediated) effects were tested using bootstrapping with bias-corrected confidence intervals.

## 3. Results

### 3.1. Descriptive Statistics

Maternal warm parenting demonstrated a mean of 22.20 (SD = 3.45). Among the EF subdomains, difficulties in planning and organization exhibited the highest mean score (M = 17.21, SD = 4.33), followed by behavioral control difficulties (M = 14.22, SD = 3.48), inattention (M = 14.88, SD = 4.13), and emotional control difficulties (M = 11.20, SD = 3.26). The mean total score for children’s problem behaviors was 49.54 (SD = 27.83) (Table 2).

Conventional criteria were applied to evaluate the normality of the study variables, whereby absolute skewness greater than 3 and absolute kurtosis greater than 10 indicate substantial departures from normality [52]. Across all variables, skewness and kurtosis values ranged from 0.04 to 1.41 and 0.23 to 2.27 in absolute value, respectively. These results fall well within acceptable limits, supporting the appropriateness of using maximum likelihood estimation in subsequent SEM.

### 3.2. Correlations Among the Variables

Pearson’s correlation coefficients revealed the associations between maternal warm parenting, the four EF subdomains, and children’s problem behaviors (Table 3). Maternal warm parenting demonstrated significant negative associations with all EF difficulties: planning–organization difficulties (r = −0.35, *p* < 0.001), behavioral control difficulties (r = −0.28, *p* < 0.001), emotional control difficulties (r = −0.29, *p* < 0.001), and inattention (r = −0.25, *p* < 0.001). Maternal warmth was also negatively associated with children’s problem behaviors (r = −0.25, *p* < 0.001), indicating that higher maternal warmth corresponds to fewer behavioral problems.

By contrast, children’s problem behaviors were positively associated with all four EF difficulties, including those related to planning–organization (r = 0.40, *p* < 0.001), behavioral control (r = 0.46, *p* < 0.001), emotional control (r = 0.50, *p* < 0.001), and inattention (r = 0.36, *p* < 0.001). These findings demonstrate that greater difficulties in EF are consistently linked to higher levels of behavioral problems among children.

### 3.3. SEM

#### 3.3.1. Measurement Model

A confirmatory factor analysis (CFA) was conducted to evaluate whether the latent constructs were adequately represented by their corresponding observed indicators. The measurement model demonstrated a good overall fit to the data, as shown in Table 4. According to established guidelines [53], values exceeding 0.90 for the NFI, CFI, and Tucker–Lewis index (TLI), together with an RMSEA value below 0.08, reflect acceptable-to-good model fit. Based on these criteria, the measurement model was judged to be appropriate and sufficiently robust for subsequent structural analyses.

#### 3.3.2. Structural Model

Considering that all fit indices of the measurement model satisfied the recommended criteria, a structural model was subsequently specified to examine whether children’s EF mediates the association between maternal warm parenting and children’s problem behaviors. Table 5 presents the SEM results. The structural model demonstrated an acceptable fit to the data, χ^2^(10) = 126.462, *p* < 0.001, with CFI = 0.971, NFI = 0.969, and TLI = 0.940—all exceeding the conventional cutoff of 0.90—alongside an RMSEA value of 0.049, indicating good overall model fit.

**Figure 2:**
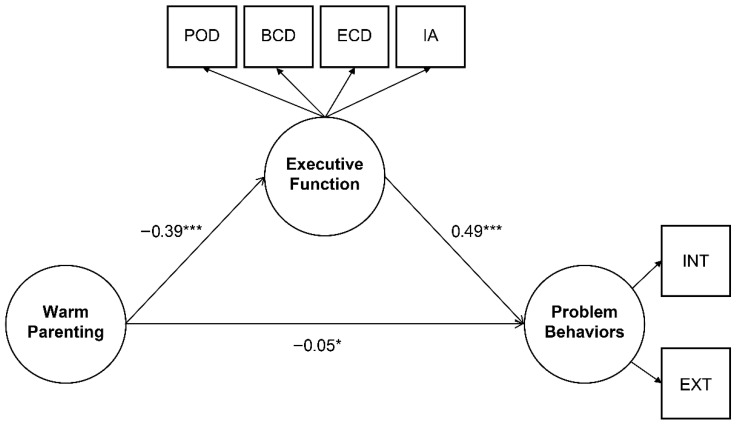
Mediating effects of executive function between parenting behaviors and children’s problem behaviors. Note: Standardized coefficients are presented. * *p* < 0.05, *** *p* < 0.001. Executive function difficulties include those related to planning–organization, behavioral control, emotional control, and inattention. POD = Planning–Organization Difficulties, BCD = Behavioral Control Difficulties, ECD = Emotional Control Difficulties, IA = Inattention, INT = Internalizing Problems, EXT = Externalizing Problems.

The parameter estimates and corresponding tests of statistical significance for both direct and indirect pathways were examined, with results summarized in Table 6 and graphically depicted in Figure 2. Maternal warm parenting exerted a significant direct effect on children’s problem behaviors (β = −0.05, *p* < 0.05). In addition, warm parenting significantly predicted children’s EF difficulties (β = −0.39, *p* < 0.001), and children’s EF, in turn, significantly predicted their problem behaviors (β = 0.49, *p* < 0.001). Furthermore, a significant indirect effect of warm parenting on children’s problem behaviors was observed through EF, providing support for the mediating role of EF (β = −0.19, *p* < 0.001).

Combined, these results indicate that higher levels of maternal warmth are associated with fewer EF difficulties in children, which subsequently relate to lower levels of problem behaviors. This pattern of effects supports the mediating role of children’s EF in the association between maternal warm parenting and children’s behavioral outcomes.

#### 3.3.3. Direct, Indirect, and Total Effects of the Structural Model

The magnitude of the direct, indirect, and total effects within the structural model was further evaluated using a bootstrapping procedure (1000 samples, 95% confidence intervals, maximum likelihood estimation). The results are summarized in Figure 2 and Table 7. Maternal warm parenting exhibited a small but statistically significant direct effect on children’s problem behaviors (β = −0.05). Further, a significant indirect effect (β = −0.19) emerged through children’s EF, indicating that warm parenting reduces problem behaviors in part by lowering children’s EF difficulties. Combined, these findings provide robust support for the mediating role of children’s EF in the pathway linking maternal warm parenting to children’s behavioral adjustment.

## 4. Discussion and Conclusions

### 4.1. Main Findings

The present study investigated how maternal warm parenting contributes to children’s behavioral adjustment, with particular attention to the mediating role of children’s EF. Using nationally representative data from the PSKC and applying an SEM framework, the current study identified both direct and indirect pathways linking warm parenting to children’s problem behaviors.

The first major finding indicates that maternal warm parenting is directly associated with fewer problem behaviors in children. This pattern is aligned with a substantial body of research showing that warm, affectionate, and responsive caregiving helps protect children from early emotional and behavioral difficulties [5,22,54,55,56,57,58]. Parenting characterized by sensitivity and emotional attunement promotes a secure relational environment that mitigates children’s arousal and reduces dysregulated behavioral expressions, thereby fostering adaptive socioemotional competencies that buffer against both internalizing and externalizing tendencies. This result also aligns with long-term evidence demonstrating that warm caregiving environments can reduce adolescents’ risk for delinquency, substance use, and other maladaptive outcomes.

The second major contribution of this study concerns the mediating role of EF. Maternal warmth was strongly associated with children’s EF skills, and EF, in turn, predicted children’s behavioral outcomes. This pattern is consistent with prior studies highlighting that emotionally supportive parenting fosters children’s regulatory capacities [42,59,60]. Longitudinal work from diverse cultural settings similarly indicates that stable, warm caregiving supports the development of attention, working memory, and self-regulation in children, particularly among those exposed to socioeconomic adversity [4,61]. From a developmental perspective, warm parenting grants children opportunities to practice decision-making, impulse control, and emotional regulation, while caregivers’ modeling of adaptive regulatory strategies further supports EF development [62]. Notably, the SEM approach enabled a clear decomposition of direct and indirect effects, revealing that the mediational pathway through EF accounted for a substantial portion of the association between warm parenting and behavioral adjustment.

EF was also strongly associated with children’s problem behaviors. Prior empirical and neuropsychological evidence has consistently shown that deficits in inhibitory control and self-regulation contribute to aggression, impulsivity, and behavioral dysregulation [3,63,64]. Difficulties in EF, particularly those reflecting prefrontal inefficiencies, may also distort children’s interpretation of social cues or lead to ineffective problem-solving strategies, resulting in maladaptive social responses [65,66]. Meta-analytic research has further highlighted that behavioral inhibition tends to exhibit especially strong associations with problem behavior [2], which is consistent with the findings of the present study considering that many indicators of the EF measure assessed inhibitory processes. Complementing prior research, the current findings also support the idea that broader regulatory disruptions—encompassing attentional and emotional control difficulties—may contribute to chronic behavior dysregulation [48].

Combined, the results indicate that maternal warmth influences children’s behavioral adjustment through both direct mechanisms and, more prominently, through its impact on EF. This underscores the importance of conceptualizing EF as not only a cognitive skill but also a relationally shaped developmental competency that enables children to navigate increasingly complex social and academic demands.

### 4.2. Educational Implications

The results of the present study indicate that EF plays a critical role in supporting children’s development in educational settings during the early elementary school years. This period is characterized by a substantial increase in demands for behavioral regulation skills. Within this context, teachers can function as not only providers of academic instruction but also emotionally supportive educators who foster children’s self-regulatory capacities through warm and responsive interactions. Such associations have been supported by prior empirical research on teacher-child interaction quality and children’s executive function in early schooling contexts [67].

In particular, clear boundary setting combined with consistent positive reinforcement from teachers may help children develop internal reference frameworks and provide effective modeling of emotional and behavioral regulation. Considering that difficulties in school adjustment during the early elementary years can have far-reaching consequences, including school refusal [68], educational interventions that support children in regulating impulsive behavior and sustaining engagement with classroom tasks are particularly important.

The educational landscape in Korea has gradually shifted away from punitive, discipline-centered approaches toward greater emphasis on student rights, early identification of difficulties, and preventive intervention. The expansion of after-school programs and targeted support for academically struggling students reflects this broader transition toward proactive support within schools. In this context, homeroom teachers of first- and second-grade students often assume roles that extend beyond academic instruction to include caregiving and socioemotional support. As demonstrated by the present findings, warm and responsive teacher–child interactions in the classroom may function as a protective factor by supporting children’s EF and thereby reducing problem behaviors. This perspective is consistent with a previous study indicating that teachers view socioemotional approaches as important for preventing and addressing problem behaviors in elementary classrooms [69].

Nevertheless, within the Korean educational context, formalized programs that systematically enhance the quality of teacher–child interactions with the explicit aim of reducing problem behaviors through EF remain limited. Although recent studies have begun examining the effectiveness and feasibility of EF training programs for clinical samples of elementary school children [70], the adaptation and implementation of such approaches within general elementary school settings represent an important direction for future educational practice and research.

### 4.3. Social Implications

From a social perspective, the present study notes that supporting parents’ caregiving capacities is a critical component of efforts to reduce children’s problem behaviors. Whereas family support policies and parent education programs have traditionally focused on caregiving skills, strategies, and attitudes, the findings of this study indicate the importance of extending such efforts to encompass parents’ emotional stability, receptiveness, and stress management capacities. Strengthening these dimensions of parenting may be particularly relevant for families experiencing high levels of caregiving stress or socioeconomic risk.

Early intervention targeting such families may play an important role in supporting children’s EF development and prevent the escalation of problem behaviors. These findings support an integrated approach that situates children’s behavioral difficulties within broader familial and social contexts.

Considering the cumulative and long-term nature of development, early elementary school years can still be considered part of an early developmental period. Therefore, identifying parenting-related difficulties during this stage and providing direct or indirect support through schools or community-based services may maximize preventive effects. From an EF perspective, children who experience difficulties with behavioral inhibition, inattention, or impulsivity may benefit from early identification through standardized screening procedures and from access to supportive interventions. Policy approaches that facilitate EF support without imposing social stigma and/or excessive financial burden may contribute to the remediation of regulatory difficulties and, in the long term, help reduce societal costs associated with persistent behavior problems.

### 4.4. Limitations

A few study limitations should be acknowledged. First, although EF was revealed to mediate the association between warm parenting and children’s problem behaviors, the relatively small magnitude of the direct effect indicates that additional contextual or individual factors may also play a role in shaping these associations. Second, the present study did not examine potential differences across sociodemographic characteristics, such as child gender, birth order, or maternal educational level, which may represent important contextual factors influencing the observed associations. Future research incorporating these variables may further refine the interpretation and applicability of the findings. Third, the reliance on maternal reports for both EF and problem behaviors increases the likelihood of shared method variance. To address this limitation, future research should consider including teacher reports, observational assessments, and/or performance-based EF tasks to strengthen the robustness of the findings. In particular, teacher reports may provide a more objective perspective on children’s behavioral regulation and social adjustment within structured classroom environments. Finally, examining higher-risk subgroups, such as children of parents experiencing depression or chronic socioeconomic hardship, will help clarify whether the protective function of warm parenting and EF generalizes across diverse contexts. Despite these limitations, this study provides novel evidence—derived from a rigorous SEM framework and a large, nationally representative sample—that EF serves as a key mechanism linking warm parenting to children’s behavioral adjustment.

### 4.5. Recommendations for Future Research

Several directions for future research emerge from the findings of the present study. First, future research should incorporate sociodemographic characteristics of the study population as potential moderating factors to more precisely examine the conditions under which the associations among warm parenting, EF, and children’s problem behaviors may differ.

Second, future studies will benefit from moving beyond reliance on a single informant by adopting varied informants combined with diverse assessment methods. For example, parenting behaviors may be assessed through maternal questionnaire reports, children’s EF through performance-based tasks or experimental observations, and problem behaviors through combined reports from both mothers and teachers. Such approaches are likely to enhance the reliability and validity of research findings.

Third, longitudinal research beyond the early school years is needed to elucidate how EF links caregiving environments to behavioral adjustment over time. Research designs that capture developmental change in later childhood and adolescence may provide a more comprehensive understanding of the conditions and processes through which EF operates as a developmental mechanism.

## Figures and Tables

**Table 1:** Sociodemographic characteristics of the participants (N = 1516).

Variable	Category	n	%
Mother’s Education	High school diploma	443	29.2
	Two-year college	421	27.8
	Four-year university	567	37.4
	Graduate school (enrolled or completed)	85	5.6
Mother’s Occupation	Office work	172	11.3
	Managerial/Professional	283	18.7
	Sales/Service	143	9.4
	Production/Labor/Manual work	52	3.4
	Other/Unemployed	866	57.1
Child’s Gender	Boy	775	51.1
	Girl	741	48.9
Child’s Age (months)	84–86 months	309	20.4
	87–88 months	697	46.0
	89–90 months	444	29.3
	91–92 months	66	4.4
Birth Order	First-born	707	46.6
	Second-born	638	42.1
	Third-born or later	171	11.3

**Table 2:** Descriptive statistics for all variables.

Variable	Min	Max	Mean	SD	Skewness	Kurtosis
Maternal Warm Parenting	6.00	30.00	22.20	3.45	−0.43	1.26
Planning–Organization Difficulties	11.00	33.00	17.21	4.33	0.56	−0.04
Behavioral Control Difficulties	11.00	33.00	14.22	3.48	1.41	2.27
Emotional Control Difficulties	8.00	24.00	11.20	3.26	1.01	0.57
Inattention	10.00	30.00	14.88	4.13	0.80	0.23
Problem Behaviors	10.00	100.00	49.54	27.83	−0.04	−1.21

Note: All skewness and kurtosis values fall within acceptable thresholds for maximum likelihood estimation. SD = standard deviation.

**Table 3:** Correlations among variables.

Variable	1	2	3	4	5	6
1. Maternal Warm Parenting	1					
2. Planning–Organization Difficulties	−0.35 ***	1				
3. Behavioral Control Difficulties	−0.28 ***	0.68 ***	1			
4. Emotional Control Difficulties	−0.29 ***	0.45 ***	0.56 ***	1		
5. Inattention	−0.25 ***	0.60 ***	0.56 ***	0.39 ***	1	
6. Problem Behaviors	−0.25 ***	0.40 ***	0.46 ***	0.50 ***	0.36 ***	1

Note: *** *p* < 0.001.

**Table 4:** Fit indices for the measurement model.

Model	χ^2^	df	χ^2^/df	NFI	CFI	TLI	RMSEA (90% CI)
Measurement model	64.913	10	6.491	0.986	0.986	0.971	0.061 (0.047–0.075)

Note: χ^2^ = chi-square, df = degree of freedom, NFI = normed fit index, CFI = comparative fit index, TLI = Tucker–Lewis index, RMSEA = root mean square error of approximation; CI = confidence interval.

**Table 5:** Fit indices for the structural model.

Model	χ^2^	df	χ^2^/df	CFI	NFI	TLI	RMSEA (90% CI)
Structural model	126.462	10	12.646	0.971	0.969	0.940	0.049 (0.040–0.058)

Note: χ^2^ = chi-square, df = degree of freedom, NFI = normed fit index, CFI = comparative fit index, TLI = Tucker–Lewis index, RMSEA = root mean square error of approximation; CI = confidence interval.

**Table 6:** Parameter estimates and significance tests for the structural model.

Path	B	β	SE	CR
Warm Parenting → Problem Behaviors	−0.07	−0.05	0.03	−2.13 *
Warm Parenting → Executive Function	−0.40	−0.39	0.03	−14.34 ***
Executive Function → Problem Behaviors	0.64	0.49	0.04	15.97 ***

Note: * *p* < 0.05, *** *p* < 0.001.

**Table 7:** Direct, indirect, and total effects in the structural equation model.

Path	Direct Effect	Indirect Effect	Total Effect
Warm Parenting → Problem Behaviors	−0.05	−0.19	−0.24
Warm Parenting → Executive Function	−0.39	—	−0.39
Executive Function → Problem Behaviors	0.49	—	0.49

## Data Availability

This study used secondary data from the nationally representative Panel Study on Korean Children (PSKC), administered by the Korea Institute of Child Care and Education (KICCE). Access to the dataset requires a formal request through the PSKC data service platform. [https://panel.kicce.re.kr (accessed on 22 January 2026)].

## References

[1] Diamond A. (2013). Executive functions. Annu. Rev. Psychol..

[2] Schoemaker K., Mulder H., Deković M., Matthys W. (2013). Executive functions in preschool children with externalizing behavior problems: A meta-analysis. J. Abnorm. Child Psychol..

[3] Quistberg K.A., Mueller U. (2020). Prospective relations between kindergarteners’ executive function skills and their externalizing and internalizing behaviors. Clin. Neuropsychol..

[4] Bernier A., Carlson S.M., Whipple N. (2010). From external regulation to self-regulation: Early parenting precursors of young children’s executive functioning. Child Dev..

[5] Eisenberg N., Zhou Q., Spinrad T.L., Valiente C., Fabes R.A., Liew J. (2005). Relations among positive parenting, children’s effortful control, and externalizing problems: A three-wave longitudinal study. Child Dev..

[6] Yeon E., Yoon H., Choi H. (2016). Structural relationship among parents’ depression, perceived family function, parenting attitudes, and young children’s internalizing and externalizing problem behaviors: Actor and partner effects. Korean J. Early Child. Educ..

[7] Im S., Im H. (2015). Structural relationships among maternal parenting variables and young children’s problem behaviors: Differences between employed and non-employed mothers. Korean J. Early Child. Educ. Care.

[8] Parmar P., Nathans L. (2022). Parental warmth and parent involvement: Their relationships to academic achievement and behavior problems in school and related gender effects. Societies.

[9] Kok R., Linting M., Bakermans-Kranenburg M.J., van IJzendoorn M.H., Jaddoe V.W., Hofman A., Verhulst F.C., Tiemeier H. (2013). Maternal sensitivity and internalizing problems: Evidence from two longitudinal studies in early childhood. Child Psychiatry Hum. Dev..

[10] Lee S.H. (2018). The effect of mother’s depression and parenting behavior on children’s problem behavior. Korean J. Child Educ. Care.

[11] Lee E.B. (2021). Relationships among subjective socioeconomic status, parental depression, negative parenting behaviors, and children’s internalizing and externalizing problem behaviors. Youth Stud..

[12] Segrin C., Givertz M., Swaitkowski P., Montgomery N. (2015). Overparenting is associated with child problems and a critical family environment. J. Child Fam. Stud..

[13] Reef J., Diamantopoulou S., van Meurs I., Verhulst F., van der Ende J. (2010). Predicting adult emotional and behavioral problems from externalizing problem trajectories in a 24-year longitudinal study. Eur. Child Adolesc. Psychiatry.

[14] Calkins S.D., Blandon A.Y., Williford A.P., Keane S.P. (2007). Biological, behavioral, and relational levels of resilience in the context of risk for early childhood behavior problems. Dev. Psychopathol..

[15] Park J., Lee K. (2020). The structural relationships among mother’s parenting stress, young children’s externalizing behavioral problems, school readiness and school adjustment in first grade. Open Parent Educ..

[16] Seo S. (2022). Effects of parenting behaviors and parenting stress on ADHD tendencies of young children. J. Korea Acad. Ind. Coop. Soc..

[17] Kochanova K., Pittman L.D., McNeela L. (2022). Parenting stress and child externalizing and internalizing problems among low-income families: Exploring transactional associations. Child Psychiatry Hum. Dev..

[18] Ha M. (2022). Effects of preschoolers’ school readiness, marital conflict, and maternal depression on externalizing problem behaviors. Hum. Soc. Sci..

[19] Gryczkowski M.R., Jordan S.S., Mercer S.H. (2010). Differential relations between mothers’ and fathers’ parenting practices and child externalizing behavior. J. Child Fam. Stud..

[20] Yeon E., Choi H. (2020). Trajectories of marital satisfaction of parent: Relatedness to behavior problems of children. J. Korea Acad. Ind. Coop. Soc..

[21] Kim D., Park Y. (2018). Maternal parenting stress, warm parenting behaviors, young children’s media use time, and externalizing behaviors: A multi-group analysis by child gender. J. Early Child. Educ..

[22] Combs-Ronto L.A., Olson S.L., Lunkenheimer E.S., Sameroff A.J. (2009). Interactions between maternal parenting and children’s early disruptive behavior: Bidirectional associations across the transition from preschool to school entry. J. Abnorm. Child Psychol..

[23] Kim S. (2011). Change in Aggression, Internalizing Problems and the Effects of Early Protective Factors: Focusing on 4th grade to 8th grade. J. Korean Child Welf..

[24] Noh Y., Kang J. (2011). The Objective and Perceived Level of Economy and Its Relationship with Mother’s Mental Health, Parenting Behaviors, and Problem Behaviors in Preschoolers. Korean J. Cult. Soc. Issues.

[25] Barnett M.A., Scaramella L.V. (2013). Mothers’ parenting and child sex differences in behavior problems among African American preschoolers. J. Fam. Psychol..

[26] Pinquart M. (2017). Associations of parenting dimensions and styles with externalizing problems of children and adolescents: An updated meta-analysis. Dev. Psychol..

[27] Hughes C., Graham A., Grayson A., Oates J. (2004). Executive function in childhood: Development and disorder. Cognitive Development.

[28] Weyandt L.L., Willis W.G., Swentosky A., Wilson K., Janusis G.M., Chung H.J., Marshall S., Goldstein S., Naglieri J. (2014). A review of the use of executive function tasks in externalizing and internalizing disorders. Handbook of Executive Functioning.

[29] Murray K.T., Kochanska G. (2002). Effortful control: Factor structure and relation to externalizing and internalizing behaviors. J. Abnorm. Child Psychol..

[30] Christopher G., MacDonald J. (2005). The impact of clinical depression on working memory. Cogn. Neuropsychiatry.

[31] Flouri E., Ruddy A., Midouhas E. (2017). Maternal depression and trajectories of child internalizing and externalizing problems: The roles of child decision-making and working memory. Psychol. Med..

[32] Hughes C., Ensor R. (2011). Individual differences in growth in executive function across the transition to school predict externalizing and internalizing behaviors and self-perceived academic success at 6 years of age. J. Exp. Child Psychol..

[33] Riggs N.R., Blair C.B., Greenberg M.T. (2004). Concurrent and 2-year longitudinal relations between executive function and the behavior of 1st and 2nd grade children. Child Neuropsychol..

[34] Utendale W.T., Hastings P.D. (2011). Developmental changes in the relations between inhibitory control and externalizing problems during early childhood. Infant Child Dev..

[35] Utendale W.T., Hubert M., Saint-Pierre A.B., Hastings P.D. (2011). Neurocognitive development and externalizing problems: The role of inhibitory control deficits from 4 to 6 years. Aggress. Behav..

[36] Friedman N.P., Miyake A., Young S.E., DeFries J.C., Corley R.P., Hewitt J.K. (2008). Individual differences in executive functions are almost entirely genetic in origin. J. Exp. Psychol. Gen..

[37] Lewis C., Carpendale J.I.M. (2009). Introduction: Links between social interaction and executive function. New Dir. Child Adolesc. Dev..

[38] Zelazo P.D. (2015). Executive function: Reflection, iterative reprocessing, complexity, and the developing brain. Dev. Rev..

[39] O’Connor T.G. (2002). Annotation: The “effects” of parenting reconsidered: Findings, challenges, and applications. J. Child Psychol. Psychiatry.

[40] Bradley R.H., McKelvey L.M., Whiteside-Mansell L. (2011). Does the quality of stimulation and support in the home environment moderate the effect of early education programs?. Child Dev..

[41] Grolnick W.S., Pomerantz E.M. (2009). Issues and challenges in studying parental control: Toward a new conceptualization. Child Dev. Perspect..

[42] Kong Y., Lim J. (2012). The effect of preschooler’s temperament and maternal parenting attitude on preschooler’s problem and prosocial behaviors -Focusing on the mediating effect of cool executive function. Korean J. Early Child. Educ..

[43] Song H. (2014). Validation of a brief self-report questionnaire for executive function difficulties among children and adolescents. Korean J. Clin. Psychol..

[44] Tomlinson R.C., Hyde L.W., Weigard A.S., Klump K.L., Burt S.A. (2022). The role of parenting in the intergenerational transmission of executive functioning: A genetically informed approach. Dev. Psychopathol..

[45] Wei W., Lu W.T., Huang M.M., Li Y. (2023). Revisiting the relationship between maternal parenting behaviors and executive functions in young children: Effect of measurement methods. Front. Psychol..

[46] Graziano P.A., McNamara J.P., Geffken G.R., Reid A. (2011). Severity of children’s ADHD symptoms and parenting stress: A multiple mediation model of self-regulation. J. Abnorm. Child Psychol..

[47] Sulik M.J., Blair C., Mills-Koonce R., Berry D., Greenberg M., The Family Life Project Investigators (2015). Early parenting and the development of externalizing behavior problems: Longitudinal mediation through children’s executive function. Child Dev..

[48] Hutchison L., Feder M., Abar B., Winsler A. (2016). Relations between parenting stress, parenting style, and child executive functioning for children with ADHD or autism. J. Child Fam. Stud..

[49] Korea Institute of Child Care and Education (KICCE) (2015). Panel Study on Korean Children (PSKC): User Guide.

[50] Cho B., Lee J., Lee H., Kwon H. (1999). Dimensions and assessment of Korean parenting style. Fam. Environ. Res..

[51] Oh K., Kim Y. (2010). Manual for the Child–Adolescent Behavior Assessment Scale.

[52] Moon S. (2009). Understanding and Application of Structural Equation Modeling.

[53] Hong S. (2000). Criteria for selecting fit indices for structural equation modeling and their rationales. Korean J. Clin. Psychol..

[54] Lengua L.J. (2006). Growth in temperament and parenting as predictors of adjustment during children’s transition to adolescence. Dev. Psychol..

[55] Lansford J.E., Sharma C., Malone P.S., Woodlief D., Dodge K.A., Oburu P., Pastorelli C., Skinner A.T., Sorbring E., Tapanya S. (2014). Corporal punishment, maternal warmth, and child adjustment: A longitudinal study in eight countries. J. Clin. Child Adolesc. Psychol..

[56] Patrick M.R., Snyder J., Schrepferman L.M., Snyder J. (2005). The joint contribution of early parental warmth, communication and tracking, and early child conduct problems on monitoring in late childhood. Child Dev..

[57] Choi M.N., Shin N. (2015). The main and interaction effects of day-care experiences and maternal parenting behavior on preschoolers’ problem behaviors. Korean J. Childcare Educ..

[58] Kim H., Park H. (2018). Moderating effects of community factors on caregiver-family factors affecting behavior problems of poor children. Korean J. Child Educ..

[59] Yoo R.H., Kim S.H. (2017). The relationship between mothers’ affective parenting and preschoolers’ peer competence: Mediating effects of preschoolers’ executive function and emotion regulation. Korean J. Child Stud..

[60] Cha M., Kim K. (2018). Effects of temperament and maternal parenting behaviors on young children’s executive function. J. Parent Educ..

[61] Hackman D.A., Gallop R., Evans G.W., Farah M.J. (2015). Socioeconomic status and executive function: Developmental trajectories and mediation. Dev. Sci..

[62] Fay-Stammbach T., Hawes D.J., Meredith P. (2014). Parenting influences on executive function in early childhood: A review. Child Dev. Perspect..

[63] Ogilvie J.M., Stewart A.L., Chan R.C.K., Shum D.H.K. (2011). Neuropsychological measures of executive function and antisocial behavior: A meta-analysis. Criminology.

[64] Rohlf H.L., Holl A.K., Kirsch F., Krahé B., Elsner B. (2018). Longitudinal links between executive function, anger, and aggression in middle childhood. Front. Behav. Neurosci..

[65] Crick N.R., Dodge K.A. (1994). A review and reformulation of social information-processing mechanisms in children’s social adjustment. Psychol. Bull..

[66] Séguin J.R. (2009). The frontal lobe and aggression. Eur. J. Dev. Psychol..

[67] Sankalaite S., Huizinga M., Dewandeleer J., Xu C., de Vries N., Hens E., Baeyens D. (2021). Strengthening executive function and self-regulation through teacher–student interaction in preschool and primary school children: A systematic review. Front. Psychol..

[68] Ulaş S., Seçer İ. (2024). A systematic review of school refusal. Curr. Psychol..

[69] Kim Y., Ryu J.-S., Lee Y.-J. (2025). Social emotional learning-based classroom behavior prevention and support for students with behavioral challenges: A focus on elementary schools. Korea Educ. Res..

[70] Mun H. (2025). Effects and applicability of an executive function enhancement program for children with ADHD. J. Behav. Anal. Support.

